# Intra- and inter-spatial variability of meiofauna in hadal trenches is linked to microbial activity and food availability

**DOI:** 10.1038/s41598-022-08088-1

**Published:** 2022-03-14

**Authors:** M. Shimabukuro, D. Zeppilli, D. Leduc, F. Wenzhöfer, P. Berg, A. A. Rowden, R. N. Glud

**Affiliations:** 1 Laboratoire Environnement Profond, REM/EEP, Institut Français de Recherche Pour L’Exploitation de La Mer, ZI de La Pointe du Diable, CS 10070, 29280 Plouzané, France; 2 Department of Biology, HADAL & Nordcee, University of Southern Denmark, Campusvej 55, 5230 Odense, Denmark; 3 National Institute of Water and Atmospheric Research, Wellington, New Zealand; 4 Helmholtz Centre for Polar and Marine Research, Alfred-Wegener Institute, Bremerhaven, Germany; 5 Department of Environmental Sciences, University of Virginia, 291 McCormick road, Charlottesville, VA 22904-4123 USA; 6 Victoria University of Wellington, Wellington, New Zealand; 7 Danish Institute for Advanced Study, University of Southern Denmark, Odense, Denmark; 8 Tokyo University of Marine Science and Technology, 4-5-7 Konan, Minato-ku, Tokyo, 108-8477, Japan

**Keywords:** Marine biology, Ecology

## Abstract

Hadal trenches are depocenters for organic material, and host intensified benthic microbial activity. The enhanced deposition is presumed to be reflected in elevated meiofaunal standing-stock, but available studies are ambiguous. Here, we investigate the distribution of meiofauna along the Atacama Trench axis and adjacent abyssal and bathyal settings in order to relate the meiofauna densities to proxies for food availability. Meiofauna densities peaked at the sediment surface and attenuated steeply with increasing sediment depth. The distribution mirrored the vertical profile of the microbial-driven oxygen consumption rate demonstrating a close linkage between microbial activity and meiofauna density. Meiofaunal standing-stock along the trench axis varied by a factor of two, but were markedly higher than values from the abyssal site at the oceanic plate. Overall, meiofaunal densities poorly correlated with common proxies for food availability such as total organic carbon and phytopigments, but strongly correlated with the microbial benthic O_2_ consumption rate. We argue that microbial biomass likely represents an important meiofaunal food source for hadal meiofauna. Observations from three trench systems underlying surface water of highly different productivity confirmed elevated meiofaunal densities at the trench axis as compared to abyssal sites on oceanic plates. Food availability appear to drive elevated abundance and variations in meiofauna densities in hadal sediments.

## Introduction

Life at great depth largely depends on the supply of organic material from the surface ocean^1–3^, and as the organic material flux decreases with increasing depth so does the density and biomass of benthic life^4–7^. This pattern is particularly true for the density and biomass of mega- and macrofauna that declines 2–3 orders of magnitude from coastal sediments to the abyssal plains^4^. The depth attenuation for meiofauna and microbial biomass is less pronounced^4^ and thus the relative importance of microbes and meiofauna for the benthic food web structure and the turnover of deposited organic material increases towards the abyssal plain^8^. Meiofauna are small (< 1000, > 20 µm) benthic metazoans providing many ecosystem services in the benthos, such as bioturbation, energy flow and nutrient cycling^9–13^. The rate of water depth attenuation for meiofauna density and biomass appear to decline or might even be reverted in the deepest oceanic regions, with hadal trench systems characterized by surprisingly high densities of living meiofauna^9,14–18^. However, trench sediments are difficult to sample and thus only few studies have investigated the structure and composition of benthic communities or the factors that regulate their distribution in the hadal realm^14,15,19–21^.

The hadal zone ranges from 6,000 m to 11,000 m depth, and is primarily comprised of 27 trench systems that stretch along tectonic subduction zones^22,23^. Here trenches form relatively narrow and long depressions in the seabed with a complex and highly variable bathymetry^22,24^. There is increasing evidence demonstrating that the central axis of the trench systems act as depocenters for organic material^25–28^ facilitated by down-slope focusing and seismic-driven mass wasting^29,30^. Recent in situ investigations have also shown that hadal trench sediments are characterized by elevated O_2_ consumption rates compared to adjacent abyssal sites at 5000–6000 m water depth^25–27,31^. The findings imply intensified biological activity in trench sediments sustained by the deposition of not only refractory, but also relatively labile and nutritious organic material^25–29^.

The higher deposition of organic material at the trench floor would be expected to be mirrored in elevated density and biomass of meiofauna^16,17,32^, but the few available assessments of the meiofauna standing stock in hadal trench settings do not provide conclusive evidence for this pattern^14,21,32^. The density and biomass of meiofauna varies by more than one order of magnitude among different trenches^15,16,33,34^, and the variability does not always correlate to the common proxies for benthic food availability such as total organic carbon (TOC) or phytodetrital material^18,32,35,36^. Living microbes could represent an important food source for meiofauna^37–39^, and cell counts or benthic O_2_ consumption, reflecting microbial activity^8^, might serve as alternative proxies for food availability in deep-sea settings.

**Figure 1:**
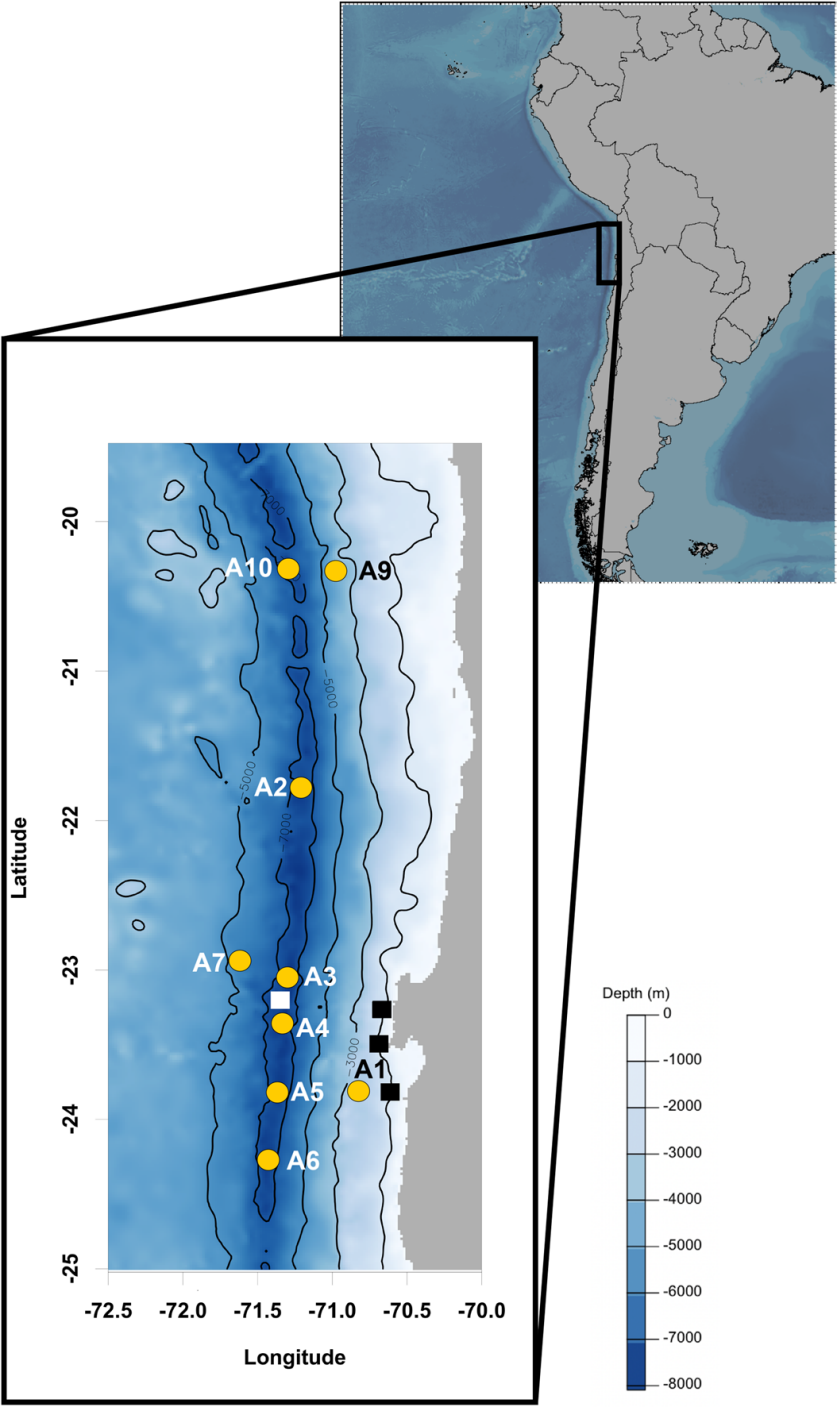
Locations of multi-corer deployments on trench axis (A2–A6 & A10), on the bathyal (A1) and abyssal (A9) sites on the continental margin, and on the oceanward abyssal plain (A7). White square Danovaro et al.^16^ hadal site; black squares Danovaro et al.^16^ bathyal sites.

The main aim of the present study is to quantify meiofaunal density and biomass in the Atacama Trench region by an extensive sampling effort targeting multiple sites along the trench axis and adjacent abyssal and bathyal sites (Fig. 1; Table 1). The Atacama Trench underlies one of the most productive oceanic regions and has been considered to be a meiofaunal hotspot with one of the highest benthic meiofaunal density and biomass reported for hadal (and deep-sea) habitats^16^. Therefore, this trench is an ideal location to examine patterns of meiofaunal distribution in relation to food availability and benthic O_2_ consumption. We also compare meiofaunal densities from the Atacama Trench to the available measurements from two other trench systems in the eutrophic Kuril-Kamchatka Trench and the oligotrophic Tonga Trench^14,15^, to evaluate whether hadal settings in general can be considered as sites of elevated meiofaunal densities.

**Table 1:** Sampling positions and water depth of the Atacama Trench sites.

Study	Site	Latitude	Longitude	Depth	Density	WW	TOC	Chl-a	OPD	DOU
				(m)	(ind. 10 cm^−2^)	(µg WW 10 cm^−2^)	(g m^−2^)	(mg m^−2^)	(cm)	(µmol m^−2^ d^−1^)
This study	A1	23˚ 48.72′ S	70˚ 50.04′ W	2560	562.6 ± 111.8	150.4 ± 20.3	490.9	5.36	1.9*	1280 ± 190
	A2	21˚ 46.86′ S	71˚ 12.48′ W	7995	509.3 ± 95.7	116.7 ± 43.5	178.7	6.7	3.2 ± 0.1	1236 ± 56
	A3	23˚ 02.94′ S	71˚ 18.12′ W	7915	646.0 ± 84.1	127.3 ± 18.2	277.6	23	2.6 ± 0.1	1793 ± 77
	A4	23˚ 21.78′ S	71˚ 20.60′ W	8085	645.5 ± 111.0	124.5 ± 36.1	216.5	17.3	3.4 ± 0.4	1490 ± 81
	A5	23˚ 49.02′ S	71˚ 22.32′ W	7770	440.4 ± 147.3	135.4 ± 36.9	214	17.2	4.0 ± 0.2	992 ± 50
	A6	24˚ 15.96′ S	71˚ 25.38′ W	7720	292.2 ± 78.6	77.0 ± 22.7	223	11.3	4.1 ± 0.3	1035 ± 143
	A7	22˚ 56.22′ S	71˚ 37.08′ W	5500	186.1 ± 93.9	42.7 ± 22.2	83.6	3.3	21.7*	355 ± 31
	A9	20˚ 19.97′ S	70˚ 58.70′ W	4050	532.1 ± 205.2	133.4 ± 41.2	224.7	15.4	6.2 ± 0.5	687 ± 101
	A10	20˚ 19.14′ S	71˚ 17.46′ W	7770	445.7 ± 93.1	110.1 ± 23.7	234.2	22.5	3.1 ± 0.3	1634 ± 71
Danovaro et al.^16^	B1	23˚ 30.50′ S	70˚ 42.80′ W	1050	550 ± 186	–	49.1 ± 17.7	35.1 ± 10.8	–	–
	C7	23˚ 15.00′ S	70˚ 40.00′ W	1355	684 ± 425	–	113.9 ± 32.8	63.9 ± 13.5	–	–
	At1	23˚ 15.00′ S	71˚ 21.00′ W	7800	6378 ± 3061	–	57.4 ± 22.7	18.0 ± 0.1	–	–

For each site is presented the average and standard deviation of meiofauna density and biomass in wet weight (WW). Total organic carbon (TOC), chlorophyll-a (chl-a), oxygen penetration depth (OPD) and the average and standard error of diffusive O_2_ uptake (DOU). The ‘*’ imply that the value is based on only one observation.

## Results

**Figure 2:**
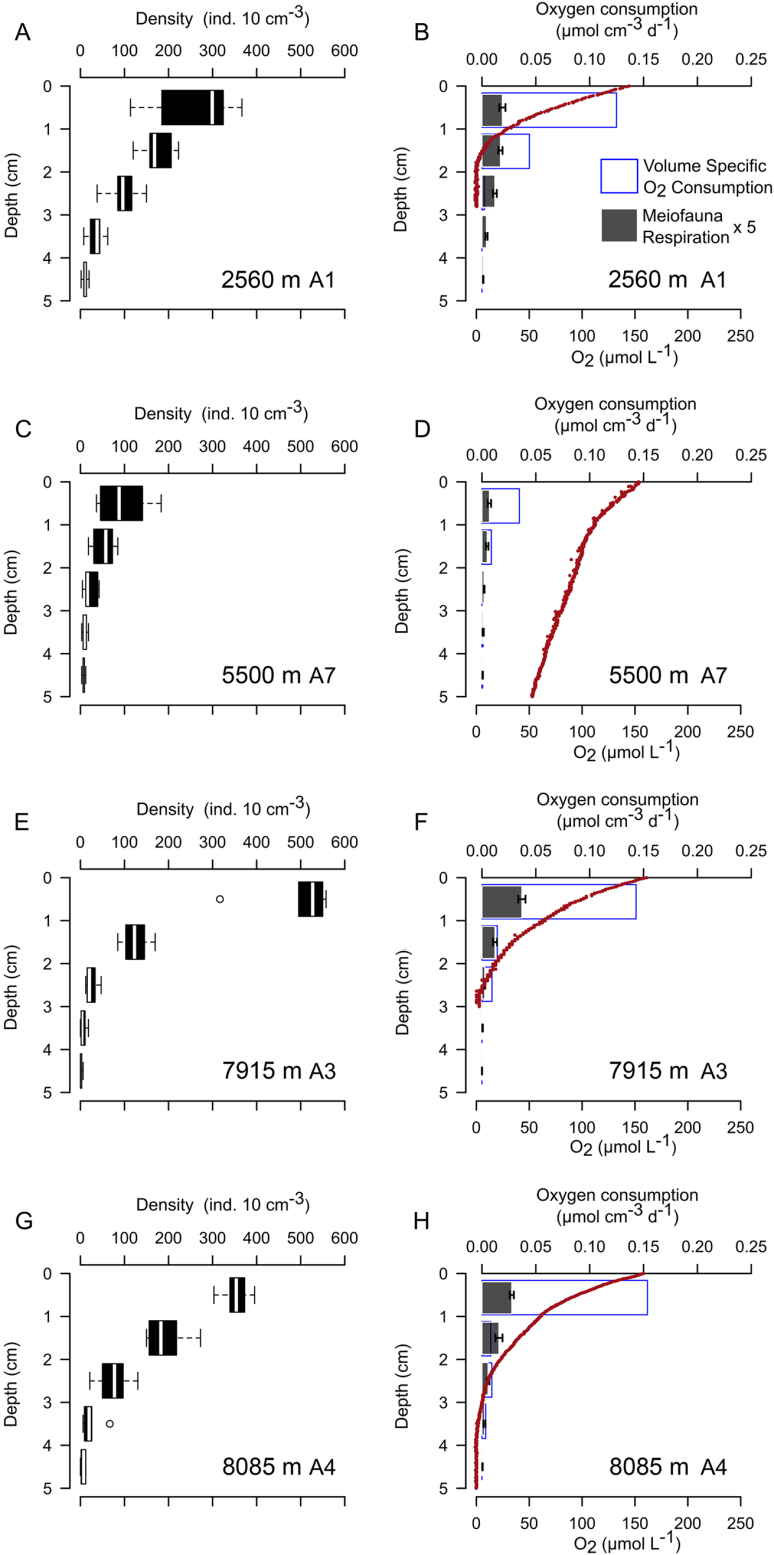
Vertical distribution of meiofauna density (**A**, **C**, **E**, **G**) and meiofauna respiration, O_2_ micro-profiles and volume specific O_2_ consumption (**B**, **D**, **F**, **H**) of four selected sites. For figures on right side: blue line bars – volume specific O_2_ consumption; grey bars – meiofauna respiration with standard errors; red dots – O_2_ micro-profiles. Values of meiofauna respiration was multiplied by 5 for better representation. Values of the volume specific O_2_ consumption is a subset of data presented in Glud et al.^26^.

The abundance of meiofauna at the bathyal (A1), abyssal (A7, A9) and hadal sites (A2, A3, A4, A5, A6, A10), was in all cases dominated by nematodes (71–94%) followed by copepods and nauplii (3–25%) and kinorhynchs (0–3%). An additional 18 taxa were identified, but they represented less than 2% of total meiofauna abundance. The benthic O_2_ penetration, measured in situ, at the bathyal site was around 1.5 cm, while values along the trench axes varied between 2.6 and 4.0 cm (Figs. 2 and S1). The O_2_ penetration at abyssal depths on both the oceanic and continental plates were considerably deeper, being more than 21.0 and 6.2 cm at site A7 and A9, respectively. The density, biomass, and the derived respiration rates of meiofauna were in all instances at their maximum at the sediment surface and attenuated steeply with increasing sediment depth. The sediment depth attenuation in meiofauna densities correlated well with the volume specific O_2_ consumption of the sediment, as calculated from the O_2_ microprofiles, and meiofauna densities reached very low values well before O_2_ was exhausted. Thus, meiofauna was in all instances nearly absent below 3 cm depth, at the bathyal, abyssal, and hadal sites (Figs. 2 and S1). Estimated meiofaunal respiration accounted for 1 to 3% of the sediment O_2_ consumption rates at the respective sites (Figs. 2 and S1). Thus, meiofauna densities clearly correlated with the microbial activity at all sites and contributed little to the benthic O_2_ consumption rate. This result was particularly prominent along the trench axis, while attenuation appeared more gradual at the bathyal (A1) and the abyssal (A9) sites at the continental plate that also might be affected by bioturbation of macrofauna communities (Figs. 2 and S1). The lowest meiofauna density, and benthic O_2_ consumption, were encountered at the abyssal plain site (A7) on the oceanic plate (Fig. 2).

**Figure 3:**
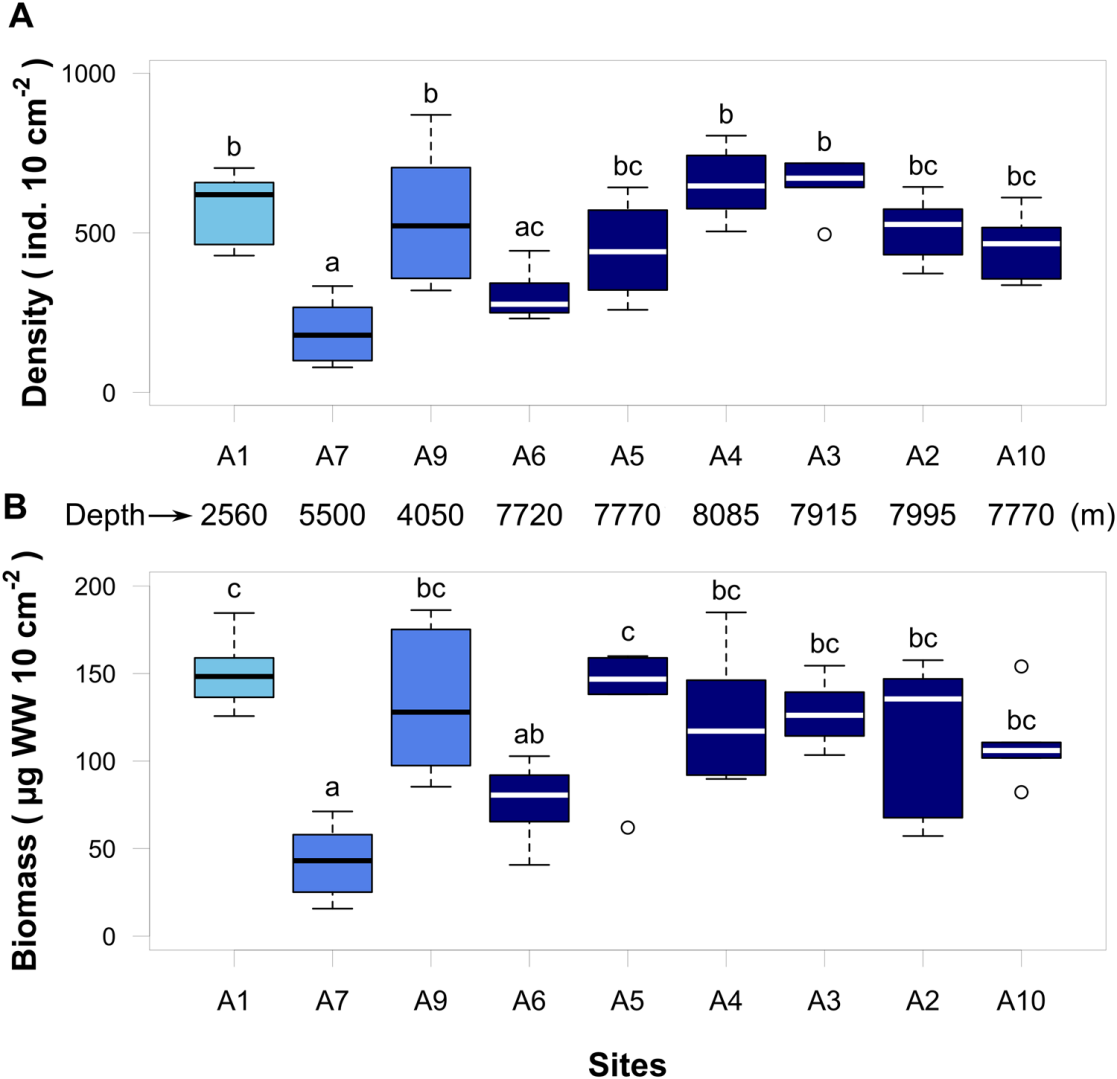
Total meiofauna density (**A**) and biomass (**B**) of integrated sediment column up to 5 cm depth. Light blue – bathyal depth; blue – abyssal depths; dark blue – hadal depths. One-way ANOVA, density – F: 9.8, *p* < 0.001, df.: 8. One-way ANOVA, biomass – F:7.0, *p* < 0.001, df.: 8. Lowercase letter above each box are results from Tukey pairwise test.

**Figure 4:**
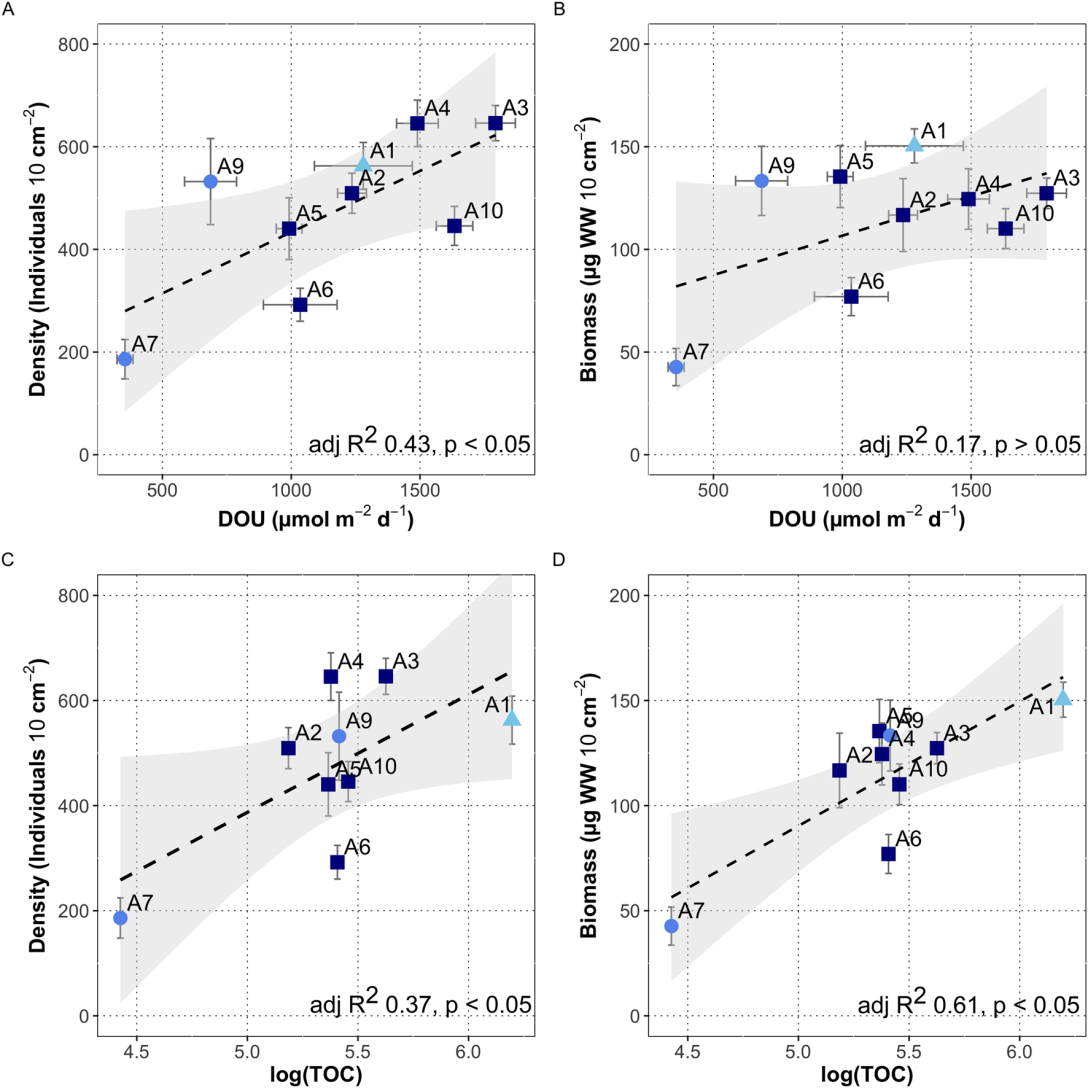
Regressions of meiofauna density/biomass against diffusive oxygen uptake (DOU) (**A**, **B**) and total organic carbon (**C**, **D**). Light blue triangle – bathyal depth; blue circles – abyssal depths; dark blue squares – hadal depths. Data of DOU is a subset of data presented in Glud et al.^26^.

The depth integrated (0–5 cm) density and biomass of meiofauna varied by twofold along the Atacama Trench axis, but were generally similar to the values encountered at the bathyal and abyssal sites on the continental plate (A1, A9) (Fig. 3, Table 1). However, values at the most southern region of trench axis (A6) were comparable with the low density and biomass found at abyssal plain site located on the oceanic plate (A7) (Fig. 3, Table 1). The depth integrated density of meiofauna significantly correlated to the benthic O_2_ consumption rates (Fig. 4A and B). Meiofauna density and biomass were also significantly correlated with sediment TOC, but the correlation was largely driven by values at the two extreme sites, i.e., the TOC-enriched bathyal site (A1) and the TOC-deprived abyssal plain site (A7) (Fig. 4D and C). Notably, there was no significant relation between the phytopigment concentrations and the abundance and biomass of meiofauna (for density adj R^2^: 0.13, *p* = 0.18 and for biomass adj R^2^: 0.02, *p* = 0.31; data not shown). Thus, it appears that meiofauna abundance and biomass, rather than being driven by the bulk TOC or phytodetrital levels, are related to the microbial activity, which presumably, is sustained by the respiration of relatively labile organic material, which may vary considerably along the trench axis.

**Figure 5:**
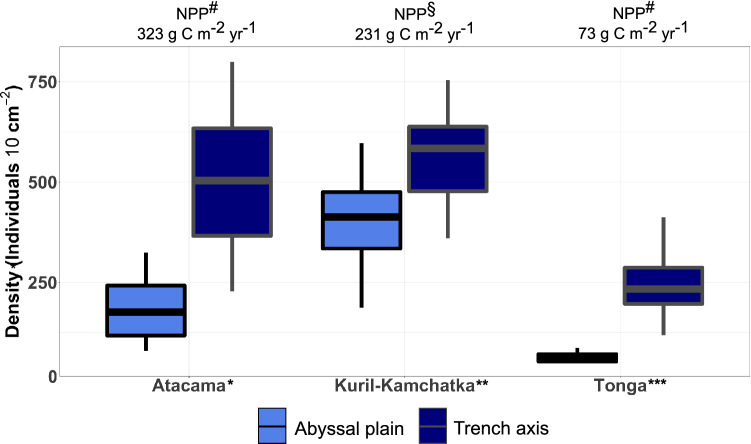
Comparison of trench axis and adjacent abyssal plain of Atacama, Japan, Kuril-Kamchatka and Tonga trenches. * this study. ** data compiled from Schimdt & Arbizu^15^, Schmidt et al.^40^, and Itoh et al.^32^ comprising: 8 abyssal plain and 7 trench axis sites. *** data compiled from Leduc et al.^14^ comprising: 1 abyssal plain and trench axis site each. All data compiled in this figure is up to 5 cm sediment depth and abyssal plain sites are on the oceanic plate. Net primary production (NPP) values was derived using Behrenfeld & Falkovski^41^ model and remote sensing data from the period 2009–2018 and previous presented in: #—Glud et al.^26^; §—Oguri et al. (in prep).

The comparison of meiofauna density up to 5 cm sediment depth from three different trench regions, clearly revealed elevated density at the trench axis as compared to the adjacent abyssal sites (Fig. 5). Moreover, meiofauna density in trench regions underlying relatively eutrophic surface waters, such as Atacama and Kuril Kamchatka trenches, was higher than values encountered in the Tonga Trench underlying relatively oligotrophic waters (Fig. 5).

## Discussion

### Distribution of meiofauna in the Atacama Trench system

With the exception of one site (A6), density and biomass of meiofauna at trench axis sites were elevated relative to values at the adjacent abyssal plain site on the oceanic plate (A7) (Fig. 3, Table 1). Values along the northern and central trench axes at water depth of almost 8,000 m were comparable to values encountered at the much shallower bathyal and abyssal sites on the continental plate (A9, A1). The findings conceptually align with previous work of Danovaro et al.^16^ in Atacama Trench, who found exceptionally high densities of benthic meiofauna at 7,800 water depth based on a single sampling event using an unusual sampling device^16^. These values are one of the highest ever reported for depositional deep-sea sediments, and are comparable to values of eutrophic coastal sites^6,10,42,43^. Danovaro et al.’s^16^ meiofaunal density values (6378 ± 3061 ind. cm^−10^) exceed, by a factor of 10, the densities observed in the present study, including the values at sites A3 and A4 located in close proximity to the latter study’s sampling site (Fig. 1, Table 1). The variability in meiofaunal standing stocks along the trench axis as encountered by the current study (~ twofold) is much smaller than the variability between our results and Danovaro et al.’s^16^ (Fig. 3, Table 1). We have no explanation for this apparent discrepancy with the findings of Danovaro et al.^16^, but confirm elevated meiofaunal densities at the trench axis as compared to the abyssal oceanic plate site.

In their model of organic matter transport in the Kermadec Trench, Ichino et al.^17^ hypothesized that higher food availabilty would be found in topographical depressions due to focusing of organic matter deposition and this would be reflected in elevated benthic faunal biomass. Here, we found maximum meiofauna densities and biomass in the deep central section of the trench while the lowest values were encountered in the wider and less steep southernmost trench region (A6, Table 1), despite similar surface ocean productivity along the trench^26^. It is, thus, plausible that variations in food availability as mediated by down-slope material focusing along the trench axis act as a prime driver for the variability in the standing stock of meiofauna in the Atacama Trench. Similar linkages have also been suggested for other hadal trench settings^15,17,32,40^.

There was no convincing link between food availability and the abundance or biomass of meiofauna, using common proxies such as TOC and phytopigment concentrations. Conversely, we saw a strong correlation to the O_2_ consumption rate of the sediment, which was mainly driven by microbial respiration. Total organic carbon is not a good indicator of labile organic material as most deposited organic carbon appear to be highly refractory and of low nutritional value^28,44,45^. Also, phytopigments can be resistant to degradation or stored in resting spores and consequently, the vertical distribution of pigments have been used to resolve deposition dynamics in coastal environments^46,47^. In contrast, the benthic O_2_ consumption is a measure of benthic community respiration and benthic mineralization^8^. Oxygen consumption at the study sites was dominated by aerobic respiration rather than oxidation of reduced constituents from anaerobic mineralization^26^ and therefore most likely reflects concurrent microbial turnover of labile organic matter. The aerobic respiration was intensified at the sediment surface, which also generally exhibited the highest microbial abundance^48^.

The derived diffusion-mediated O_2_ consumption rates presumably included contributions from meiofauna, but standard allometric relations predicted that meiofauna respiration only accounted for, at most, 3% of DOU. This value is in the lower range of other assessments in deep-sea environment that generally estimate meiofauna respiration rate to represent 1–20% of total benthic respiration^49–52^. Arguably, general allometric relations for assessing respiration for specific metazoan taxa or species are uncertain, and recently it was demonstrated that such theoretical assessments of nematode respiration rates can markedly overestimate direct meaurements on single specimens of coatal habitats^53^.

In the hadal sediment targeted in the current study the abundance of prokaryotes in the upper cm ranged between 5.7 × 10^7^ and 13 × 10^7^ cells mL^−1^^48^. Assuming a cell specific carbon content of 24.8 fg C ^54^, the prokaryotic biomass in the surface layer amounted to 1.6–4.2 × 10^−6^ gC cm^−3^. This estimate is one order of magnitude higher than the encountered meiofaunal biomass, being of the same proportion as found by Rex et al.^4^ in a wider bathymetric analysis, from 200 to 6000 m depth, of the benthic standing stock. As noted, meiofauna was mainly present in the very upper centimeter of the sediment, which expressed the highest microbial activity and prokaryotic cell numbers. In fact, the prokaryotic cell numbers generally showed a gradual decline of one order of magnitude from the sediment surface to the oxic-anoxic interface^48^, mirroring the vertical decline in meiofauna biomass.

Prokaryotic biomass represents a potentially nutritious protein- and nitrogen-enriched food source for meiofauna in marine sediments^54,55^. The carbon demand of the hadal meiofauna in the upper cm, as assessed from the estimated respiration rate and assuming a respiratory quotient of 0.85^52^, ranges from 1.33 to 7.14 × 10^−8^ gC d^−1^, which corresponds to about 0.5–4.4% of the prokaryotic biomass. These values are about six times higher than previous estimates for some deep-sea sediments^6,52^. In case the meiofauna food demand was sustained solely by the prokaryotic biomass, meiofauna predation would lead to a complete turnover of the prokaryotic communities every 25 to 200 days. The available living prokaryotic biomass would therefore be sufficient to sustain the meiofauna community. The observations align with previous studies hypothesizing that bacterial carbon could be an important resource for deep-sea nematodes^37,56^.

Marine nematodes can feed in a wide spectrum of resources and four main feeding types have been classified according to their buccal morphology^57^, later modified in agreement to their feeding behavior and to investigations based on signatures of stable carbon and nitrogen isotopes^58,59^. Due to minute and toothless buccal cavities, many nematode families are considered to be selective deposit feeders, and bacterivore^57,58^. Selective deposit feeders appear to be dominant in many deep-sea environments, including trenches^14,56,60–62^ and bacterivorous nematode species can selectively feed on different bacterial strains ensuring niche separation even if occupying the same microhabitats^39^. Gambi et al.^61^ observed that bacterivorous nematodes in Atamaca Trench region are less dominant in sites with low bacterial density and biomass. We can not discriminate the extent that the nematode feeds directly on deposited labile organic material or the prokaryotic cells. However, we see a clear correlation between the standing stock of meiofauna and the specific microbial respiration rates – both within and between the targeted sites. Given the high potential growth rate, the resolved metabolic activity, and the excessive biomass of prokaryotes, we hypothesise that the microbial community indeed represents the main food source for hadal nematodes in the Atacama Trench.

### Comparison with other trenches

Integrating values for the upper 10 cm of sediment, the first studies of meiofauna in hadal zones reported very low densities with less than 100 ind. 10 cm^−2^ in the Puerto Rico and Aleutian trenches^33,34,63^. Subsequent studies also encountered a depleted hadal meiofauna assemblage in the Ryukyu, Mariana and Kermadec trenches^19,32,64^. However, high meiofauna densities were observed in the Japan, Izu-Bonin, Kuril-Kamchatka and South Sandwich trenches, with densities of up to 1000 ind. 10 cm^−2^ encoutered at depths between 6300 and 10,900 m ^15,18,32,36,40,62^. The densities in the Atacama Trench axis found in this study are comparable to values in the Kuril-Kamchatka Trench (Fig. 5), and other trenches below relatively eutrophic surface waters, such as South Sandwich, Japan and Izu-Bonin trenches^18,36,62^ (300–1000 ind. 10 cm^−2^). On the other hand, meiofauna density of Tonga Trench axis underlying relatively oligotrophic settings is only half of the average values in eutrophic regions (Fig. 5) and similar low densities of meiofauna have also been found in Mariana and Kermadec trenches^19,64^ (between 30–50 ind. 10 cm^−2^). Meiofauna density along trench axes appeared very elevated as compared to adjacent abyssal sites at the oceanic plate, and trenches underlying productive surface oceans also displayed higher meiofauna densities than those trenches in oligotrophic regions. Thus, hadal trench axes generally appear to be sites of enhanced meiofauna densities and biomass and hotspots for deep-sea biological activity.

## Conclusion

This study showed high meiofauna densities at the sediment surface that attenuated steeply with sediment depth, mirroring the vertical profile of microbial-driven volume specific O_2_ consumption. Further, the meiofauna standing stock at the Atacama Trench axis was elevated compared to the adjacent abyssal plain site on the oceanic plate and comparable to values encountered at the nearby abyssal and bathyal sites on the continental plate. Meiofauna density and biomass along the trench axis exhibited considerable variation and were strongly correlated with benthic O_2_ consumption rates, the latter was mainly driven by microbial communities. We argue that microbial biomass is a main food source driving spatial variations of hadal meiofauna standing stocks across sediment depths within each site, among sites along the trench axis, and across the region in general. Comparing available data from three trench settings suggests that regional surface production and complex depositional processes have a strong influence on microbial activity in hadal sediments^26^, which appear to be reflected by meiofauna standing stocks.

## Methods

### Study area

Atacama Trench is situated in the Southeast Pacific Ocean off the South American continent and reaches a maximum water depth of ca. 8085 m^65^. The base of the trench is narrow (~ 2 km) in the deepest central region with a V-shape, while the trench floor towards the north and south is wider (~ 4 km) and U-shaped^66,67^. The sediment of the trench floor is dominated by silt and clay with some areas with higher contributions of sand (up to 10%)^68^. Generally, a significant fraction of the sediment along the trench axis is composed by very fine (< 1 µm) material with a thick colloidal layer^68^. The Atacama Trench is close to the Humboldt Current upwelling region with relatively high primary production (~ 910 mgC m^−2^ d^−1^)^69^.

### Sampling

Samples were collected during the expedition SO261 in 2018 on board of the *R/V* Sonne^65^. Sediment was sampled by multi-corer (MUC) (Barnett et al. 1984) at six hadal sites along Atacama Trench axis (A2, A3, A4, A5, A6, A10), two abyssal sites on each side of the trench (A7, A9), respectively and at one bathyal site (A1) (Fig. 1; Table 1). From each site, one sediment cores (id 10 cm), from two different MUC casts, were each subsampled by three smaller sub-corer with inner diameter of 2.9 cm. In total we used three subsamples per deployment comprising six sub-corers per site. The sub-cores were sliced at a depth resolution of 1 cm down to 5 cm depth and the material was fixed in 4% buffered formalin for later investigation of the meiofauna communities.

### Fauna and sediment characteristics and statistical analysis

The values and procedures to estimate the benthic concentrations of Total Organic Carbon (TOC) and chlorophyll-a (chl-*a*) have previously been presented in Glud et al.^26^ . In short, TOC was measured using 50 mg of freeze-dried sediment, based on the Rock–Eval 6 method^70^, and converted to volume specific weight from the sediment density. The chl-*a* was extracted with acetone (90%, vol:vol) and measured using a Turner fluorometer^71^. Oxygen microprofiles were measured in situ across the sediment water interphase using an autonomous benthic lander^72^. The lander was equipped with a transecting array of custom build O_2_ microelectrodes^73^ and recorded a total of 7 to 35 microprofiles at the respective sites^26^. The diffusive O_2_ uptake (DOU) and the volume specific O_2_ consumption rate were derived from the measured concentration profiles, the tortuosity corrected molecular diffusion coefficients, assuming Fickian steady-state diffusion using the public available profile interpretation software, PROFILE^74^. The microprofiles presented in the manuscript is a subset of profiles previously presented in Glud et al.^26^. Further details on procedures to obtain the values of benthic exchange and consumption rates of O_2_ are provided in Glud et al.^26^.

Meiofauna was sieved and rinsed on a 20 µm mesh size sieve. The upper mesh size used was 500 µm, but the few nematodes retained in this mesh were included in the analysis. Meiofaunal organisms retained in 20 µm were extracted from sediment by flotation in the colloidal solution, LUDOX® HS-40 and subsequent centrifugation at 3000 rpm for 10 min. The process was repeated three times. After extraction, samples were preserved in 4% buffered formalin with phloxine B, to stain the organisms alive during the moment of sampling, until sorting. Meiofauna were identified to major taxa and all animals were counted and picked out. Body volumes of all specimens were estimated from total length and maximum body width measurements using a conversion factor per meiofaunal taxa^75^. The biomass was then derived from the body volume and a presumed specific densities of 1.13^75^. Here, we present biomass data for nematodes, copepods and kinorhynchs, which together represent approximately 98% of total meiofauna encountered. The meiofauna respiration of these three groups was estimated based on allometric equation (R = 7.4 _×_ 10^–3^ * W^−0.24^, where W is the mean weight of organisms) in relation to individual dry weight^52,76^.

Spatial differences of density and biomass per site were tested using one-way ANOVA. Assumptions of normality and homogeneity of variances were tested using Shapiro–Wilk and Levene’s tests. Pair-wise post hoc comparisons were performed using Tukey honest significant differences method. Regression analysis between meiofauna density/biomass and TOC, chl-*a*, and DOU were conducted after confirming normality of the data.

## Supplementary Information

Supplementary Information 1.
