# Supplementary material for: Intra- and inter-spatial variability of meiofauna in hadal trenches is linked to microbial activity and food availability
Source: Sci Rep. 2022 Mar 14;12:4338. doi: 10.1038/s41598-022-08088-1 (PMC8921185; doi:10.1038/s41598-022-08088-1)
Supplement: Supplementary file 1 — Supplementary Information 1. [file 41598_2022_8088_MOESM1_ESM.pdf]

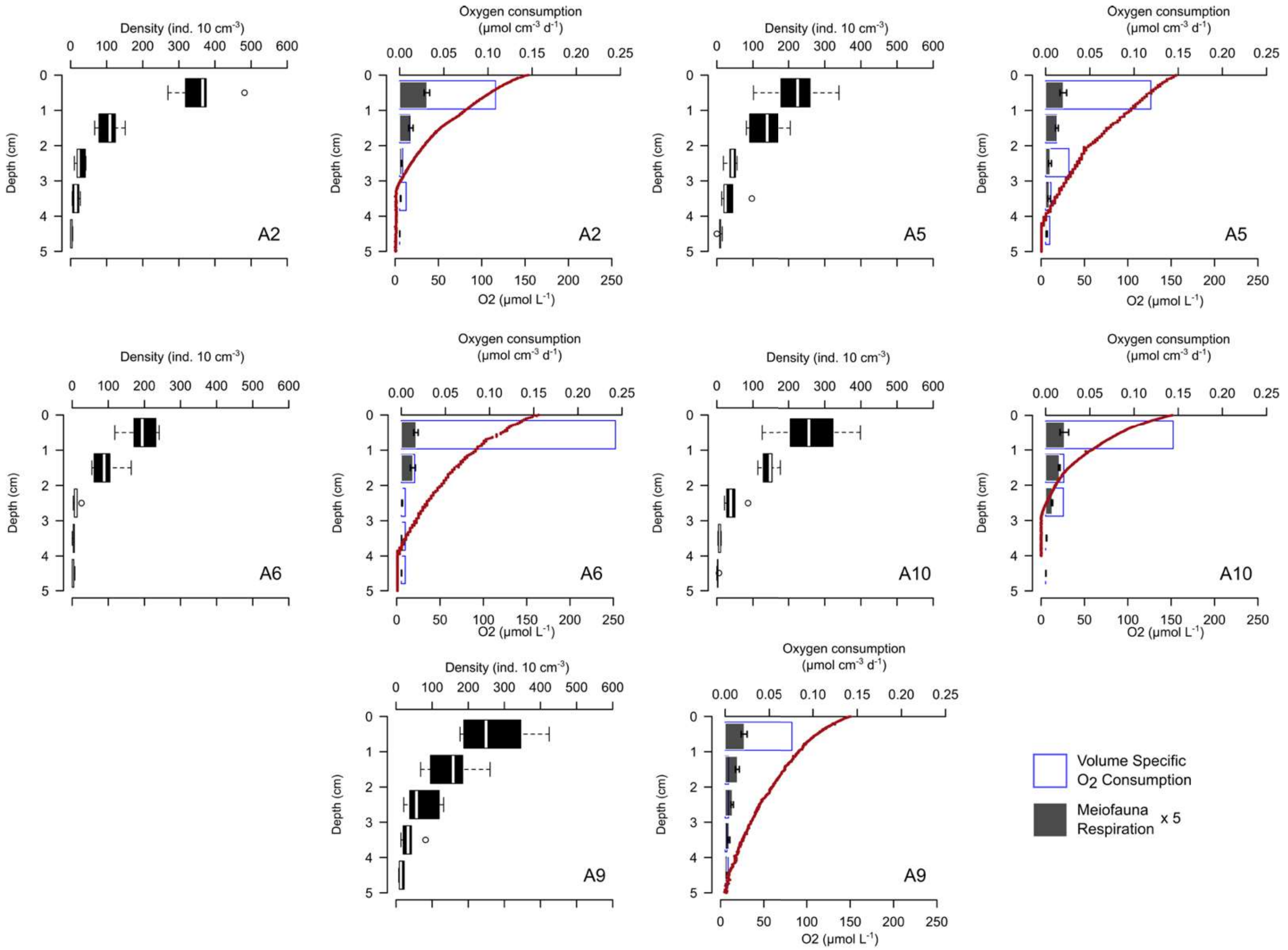

Figure S 1 - Vertical distribution of meiofauna density (plots on left side) and meiofauna respiration, O_2 micro-profiles and volume specific O_2 consumption (plots on right side). Values of meiofauna respiration was multiplied by 5 for better representation. For figures on right side: blue line bars – volume specific O_2 consumption; grey bars – meiofauna respiration with standard errors; red dots – O_2 micro-profiles. Values of the volume specific O_2 consumption is a subset of data presented in Glud et al.[26].
